# Protective Effects of Hydrogen Sulfide in Hypoxic Human Umbilical Vein Endothelial Cells: A Possible Mitochondria-Dependent Pathway

**DOI:** 10.3390/ijms140713093

**Published:** 2013-06-25

**Authors:** Yaqi Shen, Wei Guo, Zhijun Wang, Yuchen Zhang, Liangjie Zhong, Yizhun Zhu

**Affiliations:** 1 Department of Pharmacology, School of Pharmacy, Fudan University, Shanghai 201203, China; E-Mails: shenyaqi1982@163.com (Y.S.); guowei@fudan.edu.cn (W.G.); 10301030042@fudan.edu.cn (Z.W.); 071101006@fudan.edu.cn (Y.Z.); 12211030038@fudan.edu.cn (L.Z.); 2 Institute of Biomedical Sciences, Fudan University, Shanghai 201203, China

**Keywords:** HUVEC, hydrogen sulfide, hypoxia, protective effects, migration-promoting effects, mitochondria, reactive oxygen species, NaHS

## Abstract

The aim of the study was to investigate the protective effects of sodium hydrosulfide (NaHS), a H_2_S donor, against hypoxia-induced injury in human umbilical vein endothelial cells (HUVECs) and also to look into the possible mechanisms by which H_2_S exerts this protective effect. 3-(4,5-dimethylthiazol-2-yl)-2,5-diphenyltetrazolium bromide (MTT) assay and scratch wound healing assay were chosen to measure the cell viability and migration-promoting effects. The fluorescent probe, DCFH-DA and 5,5′,6,6′-Tetrachloro-1,1′,3,3′-tetraethyl-imidacarbocyanine iodide (JC-1) were applied to detect the reactive oxygen species (ROS) level and mitochondrial membrane potential (ΔΨ_m_). Furthermore, western blots were used to measure the expressions of the apoptosis-related proteins. Under hypoxic conditions, 300 μM and 600 μM of H_2_S could protect HUVECs against hypoxia-induced injury, as determined by MTT assay. Following the treatment of 60 μM NaHS for 18 h, scratch wound healing assays indicated that the scratch became much narrower than control group. After treatment with 60 μM, 120 μM, and 600 μM NaHS, and hypoxia for 30 min, flow cytometry demonstrated that the ROS concentrations decreased to 95.08% ± 5.52%, 73.14% ± 3.36%, and 73.51% ± 3.05%, respectively, compared with the control group. In addition, the JC-1 assay showed NaHS had a protective effect on mitochondria damage. Additionally, NaHS increased Bcl-2 expression and decreased the expression of Bax, Caspase-3 and Caspase-9 in a dose-dependent way. Our results suggest that H_2_S can protect endothelial cells and promote migration under hypoxic condition in HUVECs. These effects are partially associated with the preservation of mitochondrial function mediated by regulating the mitochondrial-dependent apoptotic pathway.

## 1. Introduction

In the past two decades, H_2_S, an endogenous micromolecular substance, has been widely studied by the international scientific community [1,2]. In the past 200 years, however, most studies about H_2_S, the third discovered gaseous signaling molecule in human body, have focused on its toxicity [3,4]. In 1989, Goodwin and others proposed that H_2_S possibly played a positive physiological role in the human body, thus inspiring people to realize the other side of H_2_S [5]. H_2_S has attracted considerable attention due to its multiple physiological and pathophysiological roles in various body systems. Extensive studies on the physiological functions of H_2_S have been conducted in the past two decades, and the major physiological functions of H_2_S can be summarized as follows: inducing LTP in the hippocampus through the strengthening of NMDA receptor vitality [6]; inhibiting CRH release to adjust thalamus hypophyseal-adrenal axis function [7]; activating the ATP-dependent potassium channels to relax smooth muscle cells [8]; increasing the content of intracellular glutathione for offsetting oxidative stress [1]; inhibiting the release of insulin [9–11]; acting as the endogenous regulator of immune and inflammatory response [12–14]; acting as the analgesics in gastro-intestinal tract response [15,16].

Increasing evidence indicates that H_2_S exhibits significant protective effects during a great number of cardiovascular disease states. H_2_S is endogenously synthesized from l-cysteine and this process is predominantly catalyzed by cystathionine-γ-lyase (CSE) in the cardiovascular system. Recent studies have found that CSE is not only present in vascular smooth muscles but also in vascular endothelial cells [17]. It has been shown that H_2_S protects neurons and cardiac smooth muscle from oxidative stress mainly by increasing the cellular production of GSH and preserving mitochondrial function [18,19]. Otherwise, H_2_S stimulates the endothelium-related angiogenesis and wound healing through a K_ATP_ channel/MAPK pathway [20]. Preliminary studies from our laboratory also verifies that hydrogen sulfide can promote angiogenesis [21]. In addition, it is reported that H_2_S protects different cells against chemical hypoxia-induced injury by inhibition of ROS-activated ERK1/2 pathways or p38MAPK pathways [22–25]. However, the effect of H_2_S on HUVECs during hypoxia is still unknown. Furthermore, the mechanism of a mitochondria-dependent pathway in H_2_S-induced cytoprotection have not been completely elucidated.

Therefore, cell viability and migration of HUVECs treated with H_2_S under hypoxic conditions were examined to determine the impact of H_2_S on vascular endothelial cells *in vitro*, and explore its possible functional mechanism in a mitochondrial-dependent pathway. This research offers a novel insight for future studies on the cytoprotective effects of H_2_S on HUVECs, and its treatment approaches to cardiovascular complications.

## 2. Results and Discussion

### 2.1. H_2_S Protected HUVECs under Hypoxic Conditions

**Figure 1:**
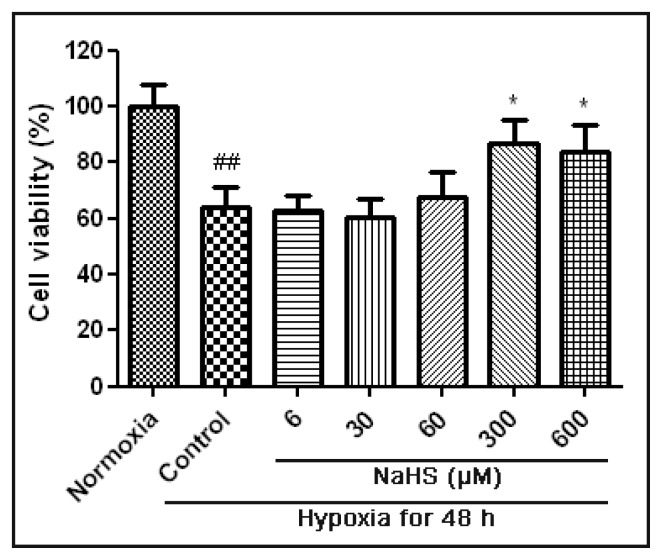
Cell viability of HUVECs subjected to different concentrations of NaHS under hypoxic condition. HUVECs were untreated or treated with 6 μM, 30 μM, 60 μM, 300 μM, and 600 μM NaHS and hypoxia for 48 h. Data are shown as mean ± SEM (*n* = 5). ^##^*p* < 0.01 *versus* Normoxia group. * *p* < 0.05 *versus* hypoxia control group.

MTT assay showed that the cell viability decreased significantly after hypoxia for 48h (Figure 1). Treated with NaHS at both 300 μM and 600 μM significantly increased the cell viability when compared with the hypoxia control group. This indicated that these two concentrations of NaHS could protect HUVECs against hypoxia-induced injury with 48 h of treatment. However, treated with different concentrations of NaHS for 6 h, 18 h and 24 h under hypoxic condition, the cell viability had no apparent change when compared with the hypoxia control group (data not show).

### 2.2. H_2_S Accelerated HUVECs Migration and Repaired the Scratch Damage under Hypoxic Condition

**Figure 2:**
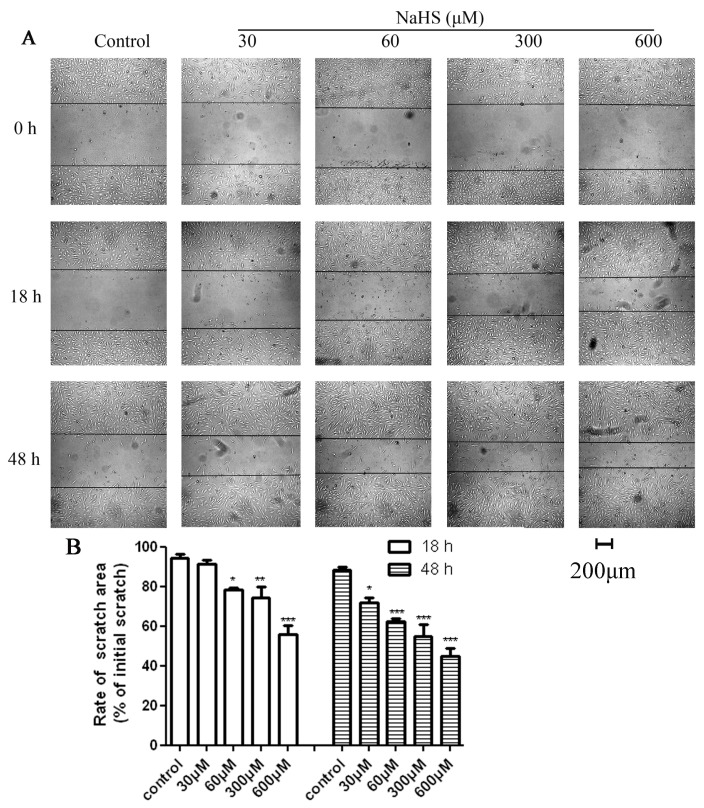
Effects of NaHS on HUVECs migration ability after different hypoxia intervals. HUVECs were untreated or treated with 30 μM, 60 μM, 120 μM, 600 μM NaHS and hypoxia for 18 h or 48 h. Cell migration ability was examined by scratch wound healing assay under a microscope. (**A**) Graphic representation of the wound closure showing changes in the wounded area; (**B**) Wound closure was evaluated by measuring the remaining scratch area and expressed as percentage of the initial scratch area. Data are shown as mean ± SEM (*n* = 3). *p < 0.05, **p < 0.01, ***p < 0.001 *versus* control.

Scratch wound healing assays indicated that NaHS could accelerate HUVECs migration and at a certain concentration, repair the damage, as shown in Figure 2. After 18 h of hypoxia, the scratches of 60 μM, 300 μM and 600 μM groups were significantly narrower than the control group. In the 30 μM group, the scratch was not significantly narrowed. However, the cells on both sides of the scratch in the 30 μM group had a migration trend. After 48 h of hypoxia, the scratches of all the medication groups appeared much narrower than the control group. According to these results, the treatment concentration of NaHS in the follow-up experiment started from 60 μM and the treatment time started from 18 h.

### 2.3. H_2_S Decreased the Concentration of Intracellular ROS

**Figure 3:**
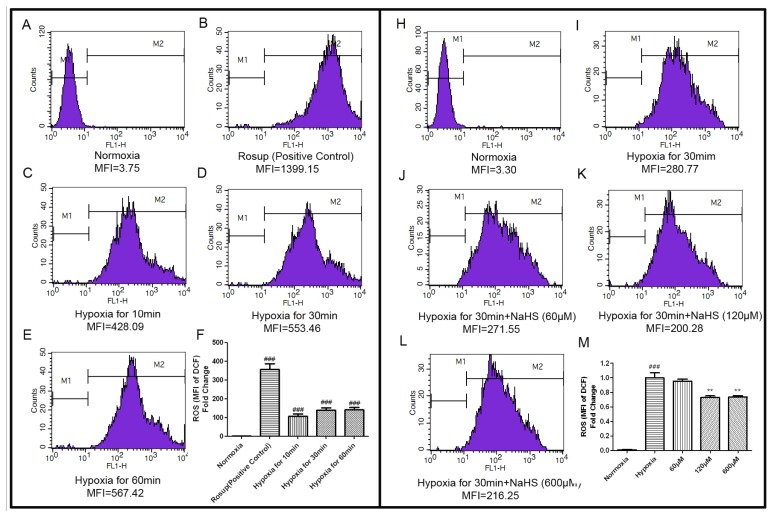
NaHS decreased the concentration of intracellular ROS. The concentration of intracellular ROS was detected by DCFH-DA fluorescent probe and a flow cytometer. (**A**–**E**) Representative pictures of DCF-derived fluorescence in HUVECs exposed to different hypoxic time measured by flow cytometer; (**F**) Quantitative analysis of the mean fluorescence intensity (MFI) of DCF. Results were expressed as the fold change compared to that of the normoxia group. Values are the mean ± SEM (*n* = 3). ^###^*p <* 0.001, *versus* normoxia group; (**H**–**L**) Representative pictures showed the effects of different concentrations of NaHS (60 μM, 120 μM, 600 μM) on ROS content in HUVECs under condition of hypoxia for 30 min; (**M**) Quantitative analysis of the mean fluorescence intensity (MFI) of DCF. Results were expressed as the fold change compared to that of the hypoxia control group. Values are the mean ± SEM (*n* = 3). ^###^*p <* 0.001, *versus* normoxia group, **p < 0.01 *versus* hypoxia control group.

Reactive oxygen species (ROS) are chemically reactive molecules containing oxygen. Excessive ROS can induce apoptosis through both the extrinsic and intrinsic pathways According to some studies [26,27], ROS concentration reaches the maximum value after 30 min of hypoxia. Therefore, the preliminary experiments tested the relationship between different hypoxia time and ROS production. Flow cytometry results presented that positive control reagents (Rosup) could increase the level of intracellular ROS in HUVECs significantly. After hypoxia for 10 min, 30 min and 60 min, the concentrations of intracellular ROS were also increased and the ROS levels were similar between hypoxia for 30 min and 60 min. Therefore, hypoxia for 30 min was taken as a follow-up experimental model (Figure 3F). After hypoxia for 30 min, different concentrations of NaHS (60 μM, 120 μM, 600 μM,), decreased the intracellular ROS levels in HUVECs obviously when compared to the hypoxia control group. The MFI decreased to 95.08% ± 5.52%, 73.14% ± 3.36%, 73.51% ± 3.05%, respectively (Figure 3M).

### 2.4. H_2_S Protected Mitochondrial Damage

**Figure 4:**
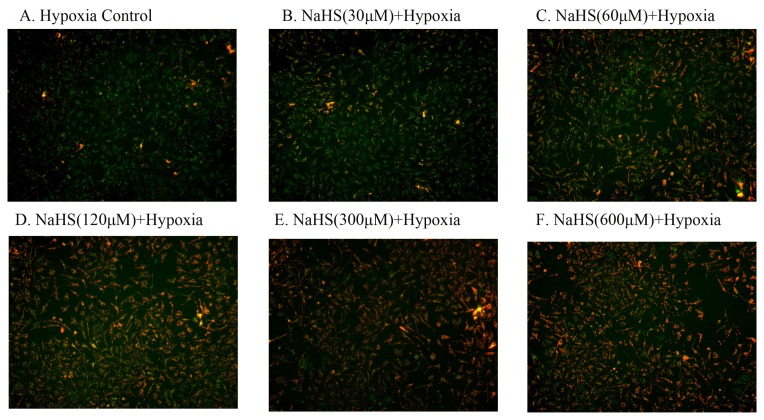

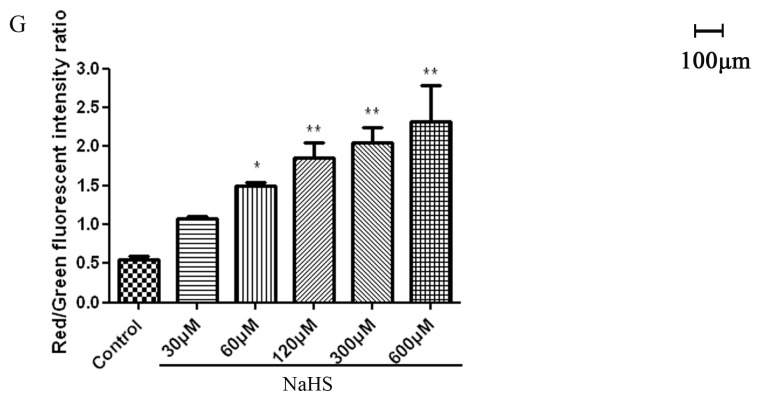
NaHS protected mitochondrial damage. (**A**–**F**) JC-1 staining. HUVECs were untreated or treated with 30 μM, 60 μM, 120 μM, 300 μM and 600 μM NaHS and hypoxia for 18 h. Mitochondrial membrane potential (ΔΨ_m_) was detected by a fluorescent dye JC-1. Red fluorescence represents the mitochondrial aggregate form of JC-1, indicating high ΔΨ_m_. Green fluorescence represents the monomeric form of JC-1, indicating dissipation of ΔΨ_m_; (**G**) Ratio of red to green fluorescence intensity, indicating ratio of JC-1 aggregate/monomer. Data are shown as mean ± SEM (*n* = 3). *p < 0.05, **p < 0.01 *versus* hypoxia control group.

Decreased mitochondrial membrane potential (ΔΨ_m_) is a sensitive indicator of mitochondrial damage. JC-1 is an ideal fluorescent probe, which is widely used for the detection of ΔΨ_m_. When ΔΨ_m_ is high, JC-1 can aggregate in the mitochondrial matrix and form a polymer (J-Aggregates), then emit red fluorescence. At lower ΔΨ_m_, the monomeric form of JC-1 cannot gather in the mitochondrial matrix and produce green fluorescence. Therefore, the change of mitochondrial membrane potential can be very easily represented by the change in the fluorescent color. Hypoxia for 18 h of HUVECs resulted in an increase in green fluorescence, indicating a loss in mitochondrial membrane potential (ΔΨ_m_) and the damage of mitochondrial (Figure 4A). The different concentrations of NaHS reduced the effects of hypoxia on mitochondrial membrane potential and increased the red fluorescence in a dose-dependent way, indicating a protective effect of NaHS (Figure 4B–F). The ratio of red and green fluorescence was also used to demonstrate the protective effect of NaHS to mitochondria (Figure 4G).

### 2.5. H_2_S Increased Anti-Apoptotic Protein Expression, and Decreased Pro-Apoptotic Protein Expression in a Dose-Dependent Way

**Figure 5:**
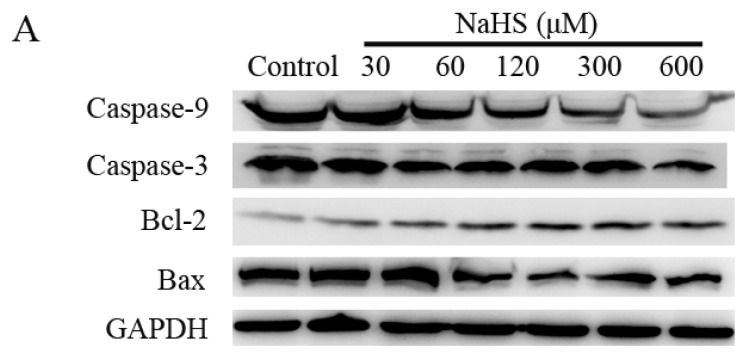

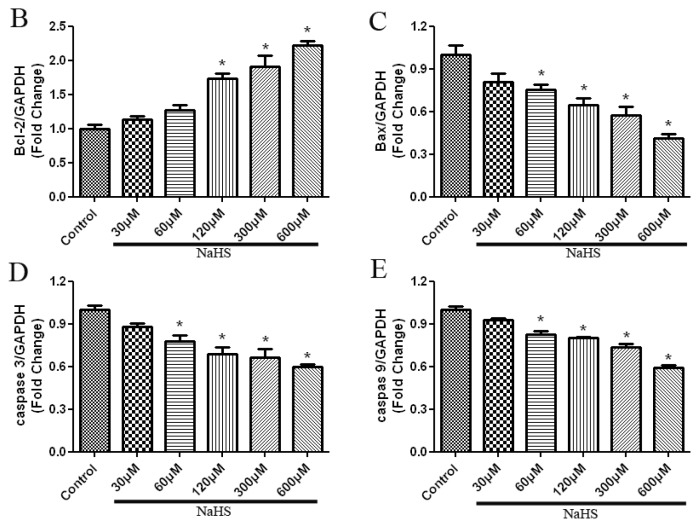
NaHS increased anti-apoptotic protein expression, and decreased the expressions of pro-apoptotic proteins in a dose-dependent way. (**A**) Representative western blot showing caspase-9, caspase-3, Bcl-2 and Bax expressions in HUVEC cells that were incubated with different concentrations of NaHS and hypoxia for 48 h; (**B**–**E**) Bar charts indicating the different intensities of apoptotic proteins between different concentrations of NaHS groups. Results were normalized against GAPDH and expressed as the fold change compared to that of the control group, respectively. Data are shown as mean ± SEM (*n* = 3). * *p <* 0.05 *versus* control group.

Western blots were used to analyze the expressions of apoptosis-related proteins in a hypoxic control group and NaHS medication groups with different concentrations. As shown in Figure 5, the Bcl-2 expression was increased, and the expression of Bax, Caspase-3 and Caspase-9 were decreased as the increase of the NaHS concentration in a dose-dependent way. These results presented statistical difference compared with the hypoxia control group.

### 2.6. Discussion

The results of the current study showed the protective effect and migration-promoting effect of H_2_S on endothelial cells under hypoxic condition. In HUVECs hypoxia model, H_2_S presented its role in the cytoprotection by increasing cell viability and promoting cell migration, as well as its antioxidation effect by reducing the production of ROS, weakening mitochondrial damage and regulating apoptotic protein expression. These findings provide evidence linking H_2_S and cytoprotection under hypoxic condition, and further, reveal the mechanisms involved in mitochondria protection by H_2_S in endothelial cells.

Vascular endothelial cells are the boundaries between circulating blood and vascular walls; they play important roles in maintaining vascular homeostasis [28]. Endothelial dysfunction is an initial factor for the occurrence and development of various cardiovascular diseases such as atherosclerosis and vasculitis [29]. Endothelial cell dysfunction is also associated with risk factors of cardiovascular diseases such as high blood pressure, high cholesterol and diabetes [29–31]. Hence, the protection of endothelial cells is a key part of the prevention and treatment of various cardiovascular diseases. Recently, multiple studies have found that a new gaseous signal molecule, hydrogen sulfide (H_2_S) can effectively protect the cardiovascular system through the following mechanisms: relaxing blood vessels [32,33], promoting the proliferation of microvascular endothelial cells and angiogenesis, inhibiting vascular matrix remodeling [34] and offsetting oxidative stress.

To clarify the protective effect of H_2_S on HUVECs during hypoxia, we firstly evaluated the toxicity of H_2_S in HUVECs. Cell viability assays indicated that H_2_S was non-toxic at micromole levels. We also observed that treatment with NaHS at the concentration of 300 μM and 600 μM could preserve endothelial cell viability. In addition, in order to expose the role of H_2_S in migration-promoting in HUVECs hypoxia model, scratch wound healing assay was performed and the results showed that when NaHS reached a concentration of 60 μM and the medicine administration time reached 18 h, NaHS could accelerate HUVECs migration and repair the damage. When the NaHS concentration increased to 300 μM or 600 μM, and the administration time reached to 48 h, the effect was more apparent. These results indicated that H_2_S has a remarkable cytoprotection and migration-promoting effect on HUVECs under hypoxic conditions.

We further explored the mechanisms of the action of H_2_S on cell injury induced by hypoxia. Although oxidation reactions are crucial for physiological functions, elevated levels of ROS can be damaging and toxic. Oxidative stress refers to the increase of oxygen radical in tissues or cells and (or) weakened absorbance capacity, resulting in the accumulated ROS in the human body or cells and the resulting oxidative damage. Our results showed that with the preliminary treatment of NaHS at a concentration of 60 μM, the average DCF relative fluorescence intensity in HUVECs was significantly lower than that of the hypoxic injury group, indicating that H_2_S could absorb intracellular ROS. Furthermore, with the increase of the concentration of H_2_S, the ROS level in hypoxic HUVECs was further reduced. This result demonstrated that H_2_S protected HUVEC cells against hypoxic injury by reducing the production of ROS.

It is reported that in the blood vessel wall, a variety of ROS will independently produce, or jointly participate in, the occurrence and development of mitochondrial damage [35]. Studies also show that the increase of ROS would directly or indirectly damage the mitochondrial membrane, thus reducing mitochondrial membrane potential (ΔΨ_m_) and promoting cytochrome c release and caspase-3 activation, which ultimately causes cell apoptosis [15,16]. In order to further probe into the functioning mechanism of H_2_S in offsetting the hypoxia-induced HUVECs damage, on the basis of previous experiment results, we observed ΔΨ_m_ and mitochondrial damage-related protein expressions to find the mechanism of exogenous H_2_S in the resistance of hypoxic injury.

It is well known that mitochondrial dysfunction is a prominent feature of apoptosis [36] as well as a notable factor associated with cell death and some models of apoptosis [37]. The formation and maintenance of normal ΔΨ_m_ is a necessary process of oxidative phosphorylation, and ΔΨ_m_ can regulate the selectivity and permeability of the mitochondrial inner membrane so as to maintain the normal structure and function of mitochondria [38]. Δψ has been reported to be involved in a variety of pathophysiological states, in particular for apoptosis [39,40]. In most apoptosis, abnormalities of the ΔΨ_m_ have been observed. Hence, we observed the impact of H_2_S on mitochondrial function. Cells were stained by JC-1 and observed under fluorescence microscopy. The results demonstrated that the preliminary treatment of NaHS with a concentration of 60 μM increased the percentage of HUVECs with red stain. This indicated that H_2_S can effectively maintain the normal potential of mitochondrial membranes and stabilize the mitochondrial function so as to prevent cell injury. As a result, it can be inferred that the protective effect of H_2_S on oxidative stress injury in HUVECs may be realized by the absorbance of ROS and the inhibition of the weakening of ΔΨ_m_.

During the revision process, a new relevant publication reported that H_2_S can inhibit H_2_O_2_ mediated mitochondrial dysfunction in human endothelial cells by preserving antioxidant defenses. Although they use a H_2_O_2_-induced hypoxia model which diffes from our hypoxia model, they obtained the similar result that H_2_S protects HUVECs against hypoxia-induced injury by preserving mitochondrial function and decreasing ROS production. They further found H_2_S protects against H_2_O_2_ induced injury of endothelial cells by reducing the deleterious effects of oxidative stress [41]. In our study, we additionally found that another possible mechanism by which oxidative stress may trigger cellular toxicity in HUVECs is the induction of the mitochondrial-dependent apoptotic pathway.

There are many apoptosis signaling pathways in cells. The mitochondrial pathway is one of the most important ways, and the Bcl-2 family of proteins is a key regulator in this pathway [42]. Bcl-2 and Bax proteins are two main members of the family. Bcl-2 is localized in the mitochondrial outer membrane, playing an anti-apoptotic role [43]. The Bax protein is similar to Bcl-2 in structure, but playing a completely opposite role. Bcl-2 functions in the outer mitochondrial membrane, in order to maintain the integrity of the membrane; On the contrary, Bax plays its role by disrupting the mitochondrial membrane integrity [44]. A study has shown that changes in the permeabilization of the outer mitochondrial membrane can cause apoptosis, and this change is directly controlled by the Bcl-2 family proteins [45]. Caspase-9 is the active enzyme upstream of the mitochondrial caspase cascade dependent apoptosis pathway; Caspase-3 is the key downstream enzyme and the final step of apoptotic pathway; it can cause DNA degradation and apoptosis by activating deoxyribonuclease when it is activated [46]. In the present study, we revealed that H_2_S increased Bcl-2 level, decreased Bax expression and inhibited the up-regulation of Caspase-3 and Caspase-9 under hypoxia condition. This finding implied that H_2_S-induced up-regulation of bcl-2 expression and downregulation of bax, Caspase-3 and Caspase-9 may be involved in the protective actions of H_2_S against hypoxia injury by preserving the structure and function of mitochondria.

## 3. Experimental Section

### 3.1. Chemicals

NaHS was used as a H_2_S donor. When it is dissolved in water, HS^−^ is released and associates with H^+^ to form H_2_S. This provides a solution of H_2_S at a concentration that is about 33% of the original concentration of NaHS [47]. NaHS is a preferred source of H_2_S as compared to other methods like direct bubbling of H_2_S gas in solutions, because NaHS can be standardized and its use allows a more accurate determination of H_2_S concentration in solution.

NaHS was purchased from Sigma-Aldrich (St. Louis, MO, USA). Dulbecco’s modified Eagle’s medium (DMEM), fetal bovine serum (FBS), and penicillin and streptomycin were purchased from GIBCO-BRL (Rrand Island, NY, USA). 2′,7′-dichlorodihydrofluorescein diacetate (H2DCF-DA) was from Molecular probes (Eugene, OR, USA). MTT (dimethyl thiazolyl tetrazolium bromide) was purchased from AMRESCO Inc. (Solon, OH, USA). Rabbit polyclonal antibodies to Bcl2, Bax, Caspase-3, and Caspase-9 were purchased from Cell Signaling Technology.

### 3.2. Cell Culture

HUVEC cell lines were cultured in DMEM supplemented with 10% fetal calf serum, 100 U/mL penicillin and 100 μg/mL streptomycin at 37 °C in the condition with 5% CO_2_ and saturated humidity.

For experiments, logarithmic phase cells were digested with 0.25% trypsin. Individual cell suspension was prepared and seeded in the appropriate plate. When cells were at a suitable confluence, they were divided into seven groups: normoxia, hypoxia, hypoxia + 30 μM NaHS, hypoxia + 60 μM NaHS, hypoxia + 120 μM NaHS, hypoxia + 300 μM NaHS and hypoxia + 600 μM NaHS.

The hypoxia model was induced as previously described by Liu *et al.* [48]. After the medicine solution with the corresponding concentration was given to the medication group, all the plates were cultured in a serum-free DMEM without glucose, and placed in an anaerobic GasPakPouch System (BD Diagnostics System, Maryland, NJ, USA) incubated at 37 °C#for 6 h, 12 h, 18 h, 24 h or 48 h. Pouches were sealed before maintained at 37 °C. Normoxia group cells were also cultured in a serum-free DMEM without glucose and incubated under normoxic conditions (5% CO_2_/95% air) in a humidified incubator at 37 °C for the same length of time.

### 3.3. MTT Assay

Cell viability was evaluated by MTT (3-(4,5-Dimethylthiazol-2-yl)-2,5-diphenyltetrazolium bromide) assay. 4 h before the administration, the original culture medium was extracted and 200 μL of serum-free DMEM was given for synchronous culturing. Then, 200 μL of the medicine solution with the corresponding concentration per well was given to the medication group, and 200 μL of serum-free DMEM medium was given to the control group. 6 complex wells were set for each group. After hypoxia for appropriate time, the medicine solution was sucked out and 200 μL of MTT (0.5 mg/mL) was added in each well and incubated for 4 h at 37 °C. After removal of MTT solution, 200 μL of DMSO was added and shaken for 15 min. The wavelength of 490 nm was selected and the absorbance value of each well was identified on Infinite M200 microplate reader. The relative survival rate of cells was calculated.

### 3.4. Scratch Wound Healing Assay

Cells were seeded in the 24-well plate at a density of 5 × 10^5^ cells/mL. When cells were at a suitable confluence, use a 200 μL pipette tip to scratch a wound through the centre of the well, kept the pipette tip vertical and do this with one lowing movement to give a clean straight edge. After treated with NaHS and hypoxia for specific time, the culture plate was removed and placed in an inverted microscope with a camera device, and for each well, 5 to 6 perspectives were selected to photograph and the results were analyzed with Image J (National Institutes of Health, Bethesda, MD, USA).

### 3.5. Assay of Reactive Oxygen Species (ROS) Generation

The concentrations of intracellular reactive oxygen species were detected using the membrane-permeable fluorescent probes 2′,7′-dichlorofluorescin diacetate (DCFH-DA) and a Cyan flow cytometer (BD FACSCalibur, San Jose, CA, USA). The DCFH-DA probe enters into cells and is hydrolyzed by intracellular esterase to form DCFH, which is trapped inside cells. The ROS can oxidize DCFH to the highly fluorescent compound 2′,7′-dichlorofluorescein (DCF) and then emit green fluorescence at 510–540 nm after excitation at 488 nm. The intracellular ROS levels were expressed as DCF relative fluorescence intensity per 10^6^ cells from independent experiments.

After treated with different concentrations of NaHS and hypoxia for 30 min, the medium in each well was extracted and added with serum-free DMEM with 10 μM of DCFH-DA and incubated for 30 min at 37 °C. The solution was shaken once per 5 min and centrifuged at 1000× *g* for 5 min. For the positive control group, positive control reagent (Rosup) were added as the ratio of 1:1000 after the incubation with the fluorescent probe, and then incubated for more 20 min. The supernatant was carefully removed and then washed with serum-free DMEM to remove the background fluorescence. The fluorescence was detected by flow cytometry with data analysis. Results were expressed as the mean fluorescent intensity (MFI) in the analyzed cells, and the MFI represents the amount of ROS.

### 3.6. Mitochondrial Membrane Potential Detection

Mitochondrial membrane potential (ΔΨ_m_) was detected by a fluorescent dye JC-1 (Beyotime, Jiangsu, China). The change from red fluorescence to green fluorescence in the JC-1 assay can be used to detect the decline in mitochondrial membrane potential. Furthermore, this transition can also be used as an early detection indicator of apoptosis. After treated with various concentrations of NaHS and hypoxia for 18 h, The HUVECs in 6-well plate was washed with PBS twice and 1 mL of serum-free DMEM medium was added, and then 1 mL of JC-1 staining working solution was added in each well. The plate was incubated for 20 min in the incubator at 37 °C with 5% CO_2_. The plate was observed and photographed under a fluorescence microscope (Carl Zeiss, Gottingen, Germany). The wavelengths of excitation and emission were 514 nm and 529 nm for detection of JC-1 monomers. 585 nm and 590 nm were used to detect JC-1 aggregates. The relative ratio of red and green fluorescence represented the change of mitochondrial membrane potential (ΔΨ_m_). Five groups of data of each well were recorded.

### 3.7. Western Blot Analysis

Cells in the large medium dish were washed with PBS and lysed on ice in RIPA buffer (Beyotime, Jiangsu, China) for 30 min. Protein concentration was measured by a BCA protein assay kit (Beyotime, Jiangsu, China). Total protein (30 μg) were separated on 10% polyacrylamide SDS gels and then transferred onto PVDF membrane, this step was followed by blocking non-specific membrane protein site in 5% skim milk solution for 2 h. Then the membrane was incubated with Polyclonal Rabbit anti- Bcl-2, Polyclonal Rabbit anti-Bax, Polyclonal Rabbit anti-Caspase-3, Polyclonal Rabbit anti- Caspase-9 (Cell Signaling Technology, Danvers, MA, USA) at 4 °C overnight. On the second day, the membranes were washed with TBST three times, then HRP-conjugated goat anti-rabbit IgG was used as a secondary antibody and incubated for 1 h at room temperature. Membranes were washed with TBST buffer, and after, an ECL ultra-sensitive light-emitting liquid was used for the chemiluminescence reaction. The signal intensity was measured by an Alpha Imager (Alpha Innotech Corp, San Leandro, CA, USA), and quantified by Quantity One software (Bio-Rad, Hercules, CA, USA).

### 3.8. Statistical Analysis

Data are expressed as mean ± SEM. Three or more treatment groups were compared by one-way ANOVA followed by post hoc analysis adjusted with a least-significant-difference correction for multiple comparisons (SPSS Inc, Chicago, IL, USA). Results were considered statistically significant when *p <* 0.05.

## 4. Conclusions

In conclusion, our results showed that H_2_S could protect HUVECs and accelerate the migration of HUVECs under hypoxic conditions. H_2_S could decrease the intracellular ROS concentration and increase the mitochondrial membrane potential of HUVECs. Furthermore, H_2_S could increase Bcl-2 expression and decrease the expression of Caspase-9, Caspase-3, and Bax under hypoxic conditions. These effects could maintain the normal structure and function of mitochondria to deal with hypoxia-induced cell injury. Our results offer new inspirations for the role of H_2_S in the cardiovascular system. Endothelial dysfunction is closely related to the occurrence of various cardiovascular diseases. Hence, it is important to conduct further studies on the impact of H_2_S in the vascular endothelial cell function. Similarly, understanding H_2_S targets and its molecular mechanism in endothelial cells can provide a solid foundation for the guidance of clinical applications.

## Abbreviations

H_2_S: Hydrogen Sulfide
NaHS: Sodium Hydrosulfide
HUVEC: Human Umbilical Vein Endothelial Cell
ROS: Reactive Oxygen Species
M: mol/L
MTT: 3-(4,5-dimethylthiazol-2-yl)-2,5-diphenyltetrazolium bromide
JC-1: 5,5′,6,6′-Tetrachloro-1,1′,3,3′-tetraethyl-imidacarbocyanine iodide
LTP: Long-Term Potentiation
NMDA receptor: *N*-methyl-d-aspartate receptor
CRH: Corticotropin Releasing Hormone
ΔΨ_m_: Mitochondrial Membrane Potential. DCFH-DA, 2′,7′-dichlorofluorescin diacetate
DCF: 2′,7′-dichlorofluorescein
MFI: Mean Fluorescent Intensity.
